# Diabetes Attitude Scale: Validation in Type-2 Diabetes Patients in Multiple Centers in China

**DOI:** 10.1371/journal.pone.0096473

**Published:** 2014-05-06

**Authors:** Qingqing Lou, Yufeng Chen, Xiaohui Guo, Li Yuan, Tao Chen, Chun Wang, Li Shen, Zilin Sun, Fang Zhao, Xia Dai, Jin Huang, Huiying Yang, on behalf of Chinese Diabetes Education Status Survey study group

**Affiliations:** 1 Jiangsu Province Hospital on Integration of Chinese and Western Medicine, Nanjing, Jiangsu Province, China; 2 Sir Run Run Shaw Hospital, Zhejiang University Medical School, Hangzhou, Zhejiang Province, China; 3 School of Nursing, Nanjing University of Traditional Chinese Medicine, Nanjing, Jiangsu Province, China; 4 Department of Endocrinology, Peking University First Hospital, Beijing, China; 5 Department of Endocrinology, West China Medical School, West China Hospital, Sichuan University, Chengdu, Sichuan Province, China; 6 Peking University First Hospital, Beijing, China; 7 Institute of Diabetes, Zhongda Hospital, Medical School, Southeast University, Nanjing, Jiangsu Province, China; 8 Department of Endocrinology, China-Japan Friendship Hospital, Beijing, China; 9 First Affiliated Hospital, Guangxi Medical University, Nanning, Jiangsu Province, China; 10 The Second Xiangya Hospital of Central South University, Changsha, Hunan Province, China; 11 The First Affiliated Hospital of Kunming Medical College, Kunming, Yunnan Province, China; Cardiff University, United Kingdom

## Abstract

**Objective:**

The aim of the paper is to report the development and psychometric testing of Diabetes Attitude Scale.

**Method:**

A prospective study was performed. The cultural equivalency and content validity of the Diabetes Attitude Scale were determined by panels of endocrinologists, physiologists, nurses and dieticians. An accurate and usable translation was obtained for each of five subscales examining attitudes on need for special training, the seriousness of type-2 diabetes, the need for controlling the condition, its psychosocial impact and the degree of autonomy given to patients in decision making. The validation was derived from 5961 patients with type-2 diabetes, recruited from 50 centers in 29 provinces throughout China between March 1st and September 30th, 2010.

**Results:**

The modified Diabetes Attitude Scale showed an acceptable level of internal consistency. The strength of the inter-correlations among the domains of five subscales suggests that the instrument measures related but separate domains of patients' attitudes toward diabetes. Moreover, the test-retest intraclass correlation coefficients were high enough to support the stability of the Chinese version of the third version of the scale.

**Conclusions:**

The psychometric properties of the Chinese version of Diabetes Attitude Scale demonstrated satisfactory validity and reliability and appeared to effectively evaluate attitudes toward diabetes in patients with type-2 diabetes.

## Introduction

Successful control of diabetes greatly depends on patients being able to manage their disease [1]. The patients can, therefore, be regarded as core team members who administer their treatment on a daily basis [2]. Studies show that diabetes patients experience various types of psychosocial and emotional problems [3], therefore, psychological factors are important to diabetes management. Many studies focusing on the effects of psychological problems on diabetes reveal that psychological factors have an impact on diabetes control [4], [5], [6]. And researchers have also developed different questionnaires to provide valuable insights in patients' view on their disease e.g. the Diabetes Distress Scale [7], [8], the Problem Areas in Diabetes Questionnaire [9], [10] or the Empowerment Scale [11], [12]. All of these established scales have been used in different studies [13], [14], [15], [16], [17], [18], [19], [20], [21], [22]. Furthermore, they have been translated into Chinese versions [23], [24], [25], [26], [27], [28], and used in China [29], [30], [31]. Attitude towards diabetes is very important. According to the attitude behavior model, a patient's intention to behave in a certain way has two major determinants, one of which is the patients' attitudes towards the behavior [32]. And it has been shown that attitudes can affect health care behavior [33], [34], [35], diabetes control and patient outcomes [36], [37]. Indeed, it has been proposed that changing patients' attitudes by monitoring the psychosocial impact of diabetes may provide a cost-effective way to improve disease control outcomes [38]. Unlike many other diseases, diabetes requires ongoing self-management of care, even when patients are asymptomatic. In contemporary research, compliance with self-management programs during the asymptomatic period has been reported to be very low, especially in key areas of diet and exercise [39]. In a wide cross-section of international type 2 diabetes patients, patient attitudes, wishes, and needs have been shown to be the foundation for successful care [40]. In Western research, several studies have produced classification systems that allow for clinical stratification of type 2 diabetes patients based on opinions and attitudes that can influence self-care behaviors [41]. It has been demonstrated, however, that diabetes patients' attitudes and opinions are highly dependent on specific cultural factors, including patients' social networks, knowledge and opinions of family and friends, and concern about the disease [33]. The rate of diabetic compliance with self-management, including insulin therapy thus varies highly by country [34], potentially as a result of variant patient opinions and attitudes towards their diabetes care. In particular, insulin adherence, a behavior known to detrimentally affect many non-compliant patients, was reported to impact patient financial situation, family and social life, and emotional well-being by the Global Attitudes of Patients and Physicians in Insulin Therapy study of 1530 insulin-treated patients, including 1350 Type 2 diabetes patients in China, France, Japan, Germany, Spain, Turkey, the UK, and the USA [4]. In developing countries, the patient population's knowledge, attitude, and practice (KAP) of diabetes is generally much worse than those in developed countries, partially due to the lack of training programs for care providers and education programs for patients [42]. Thus, many cultural factors play a role in successful diabetes care by influencing patient attitudes and opinions, and attitudes vary widely between different countries. In fact, increasing evidence suggests that improving patient educations is the most effective way to lessen the complications and costs associated with diabetes and its management [43]; however, targeted and culturally sensitive patient education programs are not possible without improved understanding of diabetic patient attitudes in these developing regions. To assess patient attitudes, we need a specific tool. The original version of the Diabetes Attitude Scale (DAS) was developed by Anderson et al in 1989. The scale which, included 31-items arranged in eight subscales, was designed to measure the attitudes of health-care professionals (HCPs) concerning important issues that affect diabetes control [44]. The third version of the scale (DAS-3) published in 1998 was designed to obtain information from patients as well as from HCPs [41]. This version collected data on 33 items within five discrete subscales and was based on the original versions of the DAS [45]. The five subscales examine patient attitudes on need for specialist healthcare training, the seriousness of type-2 diabetes, the need for controlling the condition, its psychosocial impact and the degree of autonomy given to patients in decision making. These items were chosen on the basis that they were important beliefs that were likely to predict the behavior of patients with type-2 diabetes. The items cover a range of issues relevant to the effects of diabetes on everyday life and patients' well being. However, the vase cultural differences between China and western countries make applying DAS in Chinese patients a complex task, requiring cultural consideration rather than simply translation to accurately assess psychological attitudes towards diabetes in Chinese patients.

## Materials and Methods

### Ethics Statements

The study protocol was approved by the Hospital Ethical Committee of West China Hospital, Medical School Sichuan University (approval ID: 2010(10)). Written informed consent was obtained from each patient and the primary caregivers of the minors enrolled.

### Methodology

**Figure 1:**
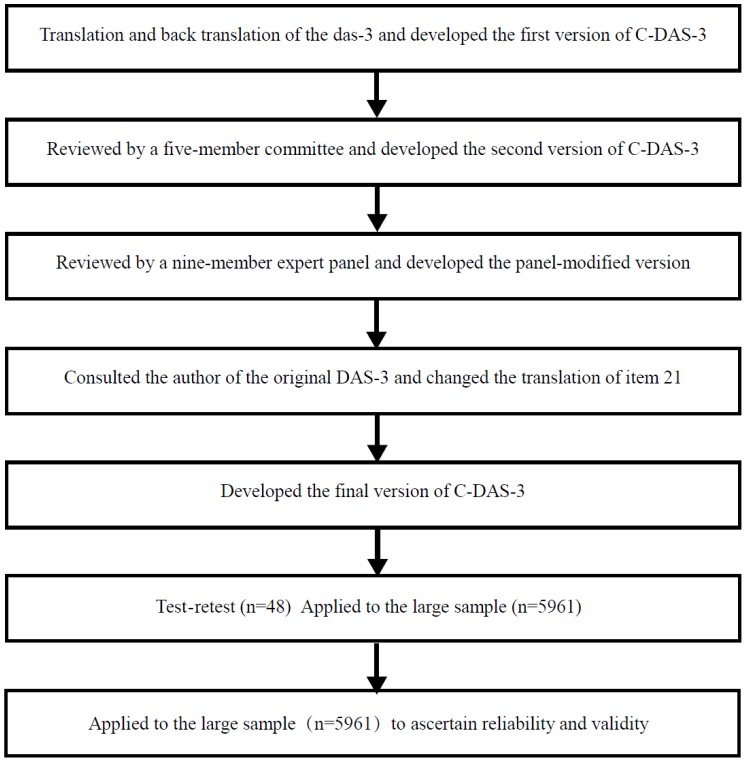
Flow diagram of the study.

The study incorporated a two-phase design (Fig. 1) that had previously been used by Shiu *et al* [27] to validate the Diabetes Empowerment Scale. This approach enabled both qualitative and quantitative assessments of the psychometric properties of the C-DAS-3 to be evaluated.

#### Phase I

Phase I included translation of the English language version of the DAS-3 into Chinese and examination of the Chinese version of DAS-3 (C-DAS-3) for cultural equivalency and content validity. The Chinese translation was guided by the Brislin's translation model [46]. A bilingual translator who was a medical doctor translated the DAS-3 into Chinese. It was then translated back into English by another physician who was also a bilingual translator. Both the original and back-translated versions were compared to determine the accuracy of the translation. The translated version (C-DAS-3) was then examined by a panel of experts comprising an endocrinologist, three diabetes nurse educators and a dietitian. The panel tested whether the translated parameters were equivalent to the original parameters and assessed whether the translated version could be readily understood and tested in a sample population of Chinese patients with type-2 diabetes.

The translated C-DAS-3 was presented to a second panel of experts for content validity assessment. This panel comprised two endocrinologists, a psychologist, five diabetes nurse specialists, and a dietician. Content validity was assessed by asking the members to rate each item as a valid measure of the construct using a three-point scale where: 1 = disagree, 2 = neutral and 3 = agree. The content validity was calculated in this manner for each item and for the overall C-DAS-3. The panel also assessed each translated item individually for accuracy, clarity and for the cultural relevance of the translation. Following minor revisions, a panel-modified version was developed and was pilot-tested in 20 patients with type-2 diabetes to check the data collection procedure and ease of understanding.

A second pilot study involving 106 patients recruited from a single center (three hospitals) in China, was undertaken to establish the psychometric properties [47] and internal consistency of the translation prior to the large scale assessment of the C-DAS-3. In this second pilot study the overall Cronbach's alpha coefficient for internal consistency was satisfactory (0.771), but the coefficient for the second dimension consistency was only 0.390. The translation was rechecked under the guidance of the original author of the DAS-3 (Dr. Anderson) and changes were made to the translation of item 21: ‘Type-2 diabetes is a very serious disease’. Content validity was repeated by the same expert panel and a final version was developed.

#### Phase II

In the second phase of the study we established the test-retest reliability, internal consistency, construct validity, and criterion validity of the final version of the C-DAS-3 in a large number of patients with type-2 diabetes.

### Study Population

The sample for Phase II was selected from among 6043 patients (> = 16 years old), who had been diagnosed with type-2 diabetes for at least 1 year. The patients were selected from 50 centers in 29 provinces in China between March 1^st^ and September 30^th^, 2010. Demographic characteristics, HbA1c, fasting and 2 h postprandial blood glucose levels were obtained from all patients. To avoid bias a single nurse from each center was trained to administer the C-DAS-3 using a defined protocol. The patients completed the questionnaires in a comfortable and quiet room. For illiterate participants the C-DAS-3 questions were read by a trained nurse and their answers were recorded.

### Instrument

The reliability of the C-DAS-3 was evaluated by estimating internal consistency using Cronbach's alpha statistic for the overall score and for each subscale. Correlations between the C-DAS-3 subscales and the criterion validity of the total C-DAS-3 score in comparison with HbA_1_c were examined using Pearson's correlation statistics, and test-retest consistency was evaluated using intraclass correlation coefficients.

The final Chinese version included translations of all 33 items in the original DAS-3. Five statements evaluated beliefs about the need for special training of healthcare staff, seven items each examined attitudes about the need for tight control of diabetes and seriousness of the disease, six items reviewed the psychosocial impact of diabetes and eight items examined what patients believed about the degree of autonomy they had in decision making. Patients indicated their agreement with each of the 33 statement as: strongly agree ( = 5), agree ( = 4), neutral ( = 3), disagree ( = 2) or strongly disagree ( = 1).

Test-retest reliability was assessed after evaluating internal consistency, in 60 patients who were willing to complete the questionnaire on two occasions 2 to 4 weeks apart. This interval was considered to be long enough for the respondents not to recall their initial answers but not too long for their attitudes to change [48].

### Statistical Procedures

SPSS version 15(IBM, USA) was used to analyze results from psychometric tests and scale analyses. In all analyses, values of *P*<0.05 were considered statistically significant. The test–retest reliability was evaluated using the paired *t*-test and the Pearson correlation coefficient. The internal consistency of the scale was examined by measuring the item reliability index, performed by calculating the Cronbach's alpha values for assessing the relatedness of each domain in the questionnaire and of each item in every domain. A principal component analysis with Varimax rotation was used for factor analysis to determine the extent of change in the questionnaire that resulted from the translation. Factors with eigenvalues ≥1.0 were included in the model.

## Results

### Demographic and Clinical Data

Eighty two of the initial 6043 questionnaires had missing data, and questionnaires from the remaining 5961 patients (98.6%, male 3233 and female 2728) were included in the analysis. The mean age was 59.5±12.4 years, the mean BMI was 24.49±4.10 kg/m^2^ and the mean duration of diabetes was 8.75±6.78 years. The majority of patients (71.2%) had at least one complication of chronic diabetes. HbA1c values were obtained from 3480 patients (Table 1).

**Table 1:** Demographic data and baseline characteristics of participants.

Character	
Sex (female), n	2728 (45.8%)
Age (mean ± SD), years	59.50±12.48
Level of education	
No formal education	365
Primary school	737
Middle school	1509
High school	1611
College level or above	1739
Diabetes duration (mean ± SD), years	8.79±6.85
Treatment, n	
Oral medication only	2069 (34.73%)
Insulin only	1399 (23.48%)
Oral medication + insulin	2269 (38.08%)
Neither oral medication nor insulin	221 (3.71%)
At least one diabetic complication, n	4238 (71.2%)
BMI (mean ± SD), kg/m^2^	24.49±4.10
HbA1c (mean ± SD; n = 3480)	8.27±2.23
<7.0	1117 (32.10%)
7.0–8.5	1086 (31.21%)
≥8.5	1277 (36.69%)
Total C-DAS-3 score (mean ± SD)	3.76±0.30

Data are expressed as means±SD or as numbers and %

(n = 5961).

### Psychometric Tests and Scale Statistics

After factor analysis, 6 factors with minimum eigenvalue of 1.0 were extracted, accounted for 59.67% of the total variance. The subscales of the original DAS could not be confirmed in the Chinese population.

Descriptive statistics for the five C-DAS-3 subscales are presented in Table 2. The mean scores ranged from 3.57±0.51 for subscale 3 (‘value of tight control’) to 4.3±0.49 for subscale 1 (‘need for special training’). The reliability (internal consistency) of the subscales based on Cronbach's alpha statistic ranged from 0.654 for subscale 5 (‘patient autonomy’) to 0.848 for subscale 4 (‘psychosocial impact of diabetes’). The comparison of Cronbach's alpha of each subscale with the original DAS-3 was also presented in Table 2. Subscale inter-correlations ranged from 0.836 for subscale 1 (‘need for special training’) versus subscale 5 (‘patient autonomy’) to 0.497 for subscale 1 (‘need for special training’) versus subscale 2 (‘seriousness of type-2 diabetes’). This high correlation between the subscales maybe partially due to the fact that the items of the subscales did not load in different factors.

**Table 2:** Descriptive statistics, comparison with the original DAS-3 and test-retest intraclass correlation coefficients for C-DAS-3.

Scale name	Number of items (N = 5961)	Mean ± SD (N = 5961)	Range (N = 5961)	Cronbach's alpha of C-DAS-3 (N = 5961)	Cronbach's alpha of DAS-3 (N = 1843)	Test-retest		
						Test (N = 48)	Retest (N = 48)	Intraclass Correlation (*P*-value)
Subscale 1	5	4.32±0.49	1.80∼5.00	0.740	0.67	4.43±0.45	4.37±0.37	0.740 (<0.0001)
Subscale 2	7	3.62±0.46	1.43∼5.00	0.706	0.80	2.88±0.44	2.68±0.35	0.657 (<0.0001)
Subscale 3	7	3.57±0.51	1.71∼5.00	0.820	0.72	2.93±0.54	2.89±0.47	0.749 (<0.0001)
Subscale 4	6	3.79±0.53	1.67∼5.00	0.848	0.65	3.30±0.52	3.19±0.59	0.865 (<0.0001)
Subscale 5	8	3.68±0.49	1.63∼5.00	0.654	0.76	3.80±0.47	3.75±0.43	0.654 (<0.0001)
Total scale	33	3.76±0.30	2.45∼4.76	0.813		3.47±0.29	3.37±0.26	0.706 (<0.0001)

DAS-3: The third version of diabetes attitude scale.

Total scale: The average score of the five subscales.

The C-DAS-3 test-retest reliability using intraclass correlation coefficients in a sample of 48 patients who completed the questionnaire on two occasions 2 to 4 weeks apart, was 0.82 (95% CI: 0.68–0.89). The coefficients between the two repeat tests among the same subscales ranged from 0.66 for subscale 5 (‘patient autonomy’) to 0.87 for subscale 4 (‘psychosocial impact of diabetes’) (Table 2). Paired t-test was also applied to evaluate the test-retest reliability which shows no statistical difference between the test and re-test (*t* = 0.18, *p* = 0.857).

The norms of the C-DAS-3 in the diabetes patients whose HbA1c, fasting and 2 h postprandial blood glucose levels were successfully controlled were 3.79±0.29, 3.79±0.30, and 3.79±0.29 respectively.

The correlation coefficient between the total Chinese version of Diabetes Attitude Scale score and HbA1c was −0.040 (*p* = 0.018), and weak correlations between 4 subscales (subscale 1∼subscale 4) and A1c were also found. The correlation coefficients were −0.033 (*p* = 0.042), −0.047 (*p* = 0.006), −0.077 (*p* = 0.000), −0.066 (*p* = 0.000), and −0.024 (*p* = 0.153), respectively.

There were correlations between the C-DAS score and age, education level, duration of diabetes, presence of complications, and accepted diabetes education, but there was no association between the Chinese DAS score and gender, insulin treatment, and BMI. (Table 3)

**Table 3:** Correlations between C-DAS-3 and demographic/medical variables.

	Total scale of C-DAS-3	Pearson's r	Spearman	Mann-Whitney U	*P*-value
**Gender**				no number	0.101
**Age**		−0.164			0.000
**Diabetes duration**		0.274			0.000
**BMI**		0.003			0.862
**A1c**		0.040			0.018
**Education level**			0.352		0.000
**Complications**			0.387		0.000
**Diabetes Education**			0.226		0.000
**Insulin treatment**				−1.213	0.225

## Discussion

### Theoretical Framework

Based on the results presented here, the C-DAS-3 is a validated tool that can be used in the Chinese population with type-2 diabetes. Our findings provide support for the construct validity and test-retest reliability of the C-DAS-3. The internal consistency of the scale was 0.813, which was validated by previously proposed theoretical frameworks [49], [50]. The strength of the inter-correlations among the domains of five subscales suggests that the instrument measures related but separate domains of patients' attitudes toward diabetes. Moreover, the test-retest intraclass correlation coefficients were high enough to support the stability of the C-DAS-3.

### Psychosocial Impact, Training, and Patient Well-Being in China

The mean nurse evaluated subscale scores for the C-DAS-3 were all somewhat lower than that in the original DAS-3 (subscale 1: 4.23 versus 4.67; subscale 2: 3.62 versus 4.58; subscale 3: 3.57 versus 4.43; subscale 4: 3.79 versus 4.39; subscale 5: 3.68 versus 4.33) [45]. The reliabilities (‘internal consistency’) of the subscale 1, subscale 3 and subscale 4 of the C-DAS-3 were higher than that in the original DAS-3 (subscale 1: 0.74 versus 0.67; subscale 3: 0.820 versus 0.72; subscale 4: 0.848 versus 0.65) [45] but others of the C-DAS-3 were lower than that in the original DAS-3 (subscale 2: 0.706 versus 0.80; subscale 5: 0.654 versus 0.76) [45]. The results were comparable. Cultural difference is the possible reason for different reliability outcomes in the Chinese and in the western populations. For example, the reliabilities of the subscale 3 (value of tight control) in the C-DAS-3 was higher than that in the original DAS-3. Compared with western patients, Chinese patients are more stressed on value of tight glycemic control, therefore patients tended to give a more homogeneous response pattern in the Chinese version of the DAS tight control than in a western population. In America, diabetes education has evolved from primarily didactic presentations to more theoretically based empowerment models [51] emphasizing on patient autonomy. Therefore Americans tended to give a more homogeneous response pattern in the original version of the DAS patient autonomy. That is why the reliabilities of the subscale 5 (patient autonomy) in the C-DAS-3 was lower than that in the original DAS-3.

Both the original and Chinese versions of DAS-3 have validated by the experts, but in the factor analysis, the subscales of the original DAS could not be confirmed in the Chinese population, this may be due to the culture difference, diabetes education qualities, as well as the education levels of the participants between two countries.

We found that the highest inter-correlations were for the relationships between the psychosocial impact of diabetes (subscale 4; 0.768) and the need for special training (subscale 1) and between patient autonomy (subscale 5; 0.836) and the need for special training (subscale 1). These findings suggest that patients' sense of wellbeing may be related to the degree of specialist training received by medical professionals. However, in the original DAS-3 the highest inter-correlation (0.63) was between the seriousness of diabetes and the need for tight control.

In our study, we found correlations between the C-DAS score and age, education level, duration of diabetes, presence of complications, and accepted diabetes education. Patients with higher education level and those who received diabetes education have more serious attitude on diabetes. On the other hand, younger patients (usually with higher education degrees), and patients with complications and longer diabetes duration take diabetes more seriously.

In China, attitudes towards diabetes have rarely been explored, though limited reports of attitudes towards diabetes complications have been reported. In a study of diabetic glaucoma in rural Chinese patients, Yan *et al*. [52] reported that misconceptions about the nature of the disease commonly results in poor adherence to routine examination schedules during asymptomatic periods. Furthermore, it was proposed that patient education by trained nurses should be implemented through home contact to improve patient compliance [52]. Similar results were demonstrated in a wide cross-section of international diabetic patients by the Diabetes Attitudes, Wishes, and Needs (DAWN) study, which indicated that patient compliance with self-management behaviors, particular diet and exercise, was as low as 2.9% in Type 2 diabetic patients [53]. The findings of the current research combined with these previous indications highlights the need for both improved nursing training that includes specialization in diabetes care and patient education programs, particularly in under-served and rural regions of China.

### Limitations

The study is to some degree limited by the relatively small populations used to pilot the questionnaire and identify areas that needed changing in the final version, and also by the relatively small population (48 out of 60 patients) available to evaluate the test-retest reliability, it means only 80% of the patients completed the second questionnaire, therefore, there is a selection bias. It would also be beneficial to compare the validity of this version of the C-DAS-3 in other provinces and regions of China.

Only a low level of statistical evidence for a negative relationship between overall C-DAS-3 scores and HbA1c levels was found (r = −0.04). It is theoretically possible that the statistical significance of this correlation may have been due to the large sample size without signifying any clinical significance or importance. However, a previous study undertaken in Argentina study provided evidence to suggest that changing the attitudes of diabetic patients through reeducation contributed to improved care and quality of life and decreases the financial burden of the disease [54]. The attitudes of Chinese patients may be similarly improved through education programs. Before education programs can be designed, however, there is an urgent need for better assessment of attitudes of type-2 diabetes patients, as provided in the current study. Thus, this research provides an essential first step to improving care for diabetes patients in China and potentially implementing education programs in the future.

In conclusion, psychometric properties of a translated version of the C-DAS-3 demonstrated satisfactory validity and reliability and provided an effective measure for evaluating attitudes toward diabetes in a Chinese population with type-2 diabetes. This assessment requires further validation in other populations of Chinese patents with diabetes.
